# Application of deep learning for bronchial asthma diagnostics using respiratory sound recordings

**DOI:** 10.7717/peerj-cs.1173

**Published:** 2023-01-10

**Authors:** Theodore Aptekarev, Vladimir Sokolovsky, Evgeny Furman, Natalia Kalinina, Gregory Furman

**Affiliations:** 1 Physics Department, Ben-Gurion University of the Negev, Be’er Sheva, Israel; 2 Department of Faculty and Hospital Pediatrics, Perm State Medical University named after Academician E. A. Wagner, Perm, Russia; 3 Education Department, Tel-Hai College, Tel-Hai, Upper Galilee, Israel

**Keywords:** Bronchial asthma, Respiratory sound, Deep learning, Computer-assisted diagnostics, Database

## Abstract

Methods of computer-assisted diagnostics that utilize deep learning techniques on recordings of respiratory sounds have been developed to diagnose bronchial asthma. In the course of the study an anonymous database containing audio files of respiratory sound recordings of patients suffering from different respiratory diseases and healthy volunteers has been accumulated and used to train the software and control its operation. The database consists of 1,238 records of respiratory sounds of patients and 133 records of volunteers. The age of tested persons was from 18 months to 47 years. The sound recordings were captured during calm breathing at four points: in the oral cavity, above the trachea, at the chest, the second intercostal space on the right side, and at the point on the back. The developed software provides binary classifications (diagnostics) of the type: “sick/healthy” and “asthmatic patient/non-asthmatic patient and healthy”. For small test samples of 50 (control group) to 50 records (comparison group), the diagnostic sensitivity metric of the first classifier was 88%, its specificity metric –86% and accuracy metric –87%. The metrics for the classifier “asthmatic patient/non-asthmatic patient and healthy” were 92%, 82%, and 87%, respectively. The last model applied to analyze 941 records in asthmatic patients indicated the correct asthma diagnosis in 93% of cases. The proposed method is distinguished by the fact that the trained model enables diagnostics of bronchial asthma (including differential diagnostics) with high accuracy irrespective of the patient gender and age, stage of the disease, as well as the point of sound recording. The proposed method can be used as an additional screening method for preclinical bronchial asthma diagnostics and serve as a basis for developing methods of computer assisted patient condition monitoring including remote monitoring and real-time estimation of treatment effectiveness.

## Introduction

Bronchial asthma (BA) is one of the most common chronic diseases in all countries regardless of their level of development. Diagnostics of BA is based on complex examination of patients including methods such as assessment of the lungs’ functional state, lung auscultation by doctors as well as analysis of anamnesis and historic family health (GINA, 2022; Gaillard et al., 2021). In some cases, X-ray examination and computer tomography are utilized to carry out differential diagnostics and exclude other lung diseases (Ash & Diaz, 2017). One of the basic objectives of asthma treatment is prevention of severe acute conditions that require permanent monitoring (including remote) of patient’s condition (GINA, 2022; Gaillard et al., 2021). Fast and objective assessment of the efficiency of medications prescribed to a patient is also important. Lung function assessment and imaging examination are difficult to perform in certain cases of pediatric practice, especially for children under 5 years old. Most commonly, such children cannot adequately participate in investigations of the functional state of lungs. Results of physical examination using lung auscultation by doctors are to a considerable degree subjective (Bahoura & Lu, 2006; Brand et al., 2008). During auscultation, doctors diagnose the presence of pathological respiratory sounds. However, multiple different lung diseases can be accompanied by the same pathological respiratory sounds (GINA, 2022; Furman et al., 2020; Sovijarvi et al., 2000). Computer-assisted analysis of breath sounds can become an additional method of preclinical diagnostics of lung diseases, including BA in children; it can also be an instrument for objective express diagnostics of the disease and remote monitoring of the state of patient’s health, becoming a component of telemedicine (Furman et al., 2014; Furman et al., 2015; Furman et al., 2021; Furman et al., 2020; Chekhovych et al., 2018 and references therein). Computer-assisted methods of lung disease diagnostics are free of subjectivity and enable the ability to analyze changes in respiratory sounds, which cannot be detected by the human ear. The reviews (Chekhovych et al., 2018; Kim et al., 2021) discuss the main methods of respiratory sound processing including methods of machine learning and deep learning that can be used for development of computer-assisted diagnostic software applications. In comparison with methods and models that analyze patient’s state, the models that utilize machine learning are often less interpretable but demonstrate higher accuracy of diagnostic predictions^1^ 1At the time of writing this article, in English literature devoted to machine learning, the term “prediction” is gradually being replaced by the term/neologism “inference”. The term “inference” is more accurate as it mystifies the work of artificial neural networks to a lesser degree. Further, in this work we will use both terms.(Elmarakeby et al., 2021; Shrikumar, Greeside & Kundaje, 2017; Murdoch et al., 2019). Despite many works devoted to development of computer-assisted diagnostics methods, a method suitable for application in real hospital conditions has not yet been developed (Chekhovych et al., 2018).

In this study, we have developed methods of computer-assisted diagnostics of respiratory diseases that utilize deep learning techniques to analyze respiratory sound recordings. In this study the main attention is given to the development of BA diagnostics methods. When developing these methods, we have used the database described in the next section.

Computer-assisted analysis of breath sounds was performed at Ben-Gurion University of the Negev (Israel). Software was implemented in Python programming language using numpy (Harris et al., 2020), librosa (McFee et al., 2021), scipy (Virtanen et al., 2020), scikit-learn (Pedregosa et al., 2011), and PyTorch (Paszke et al., 2019) libraries as well as the Python standard library. All tools used to develop the software are open-source software; they are distributed under and protected by the permissive licenses.

## Database

The anonymous database that we formed contains the following basic characteristics of a person under examination: age, gender, health status information, diagnosis, links to files of digital audio recordings of respiratory sounds, time of recording, and recording point (see below). The database contains respiratory sound recordings of healthy volunteers and patients with asthma and other respiratory diseases. The health status of asthmatic patients was classified into exacerbation, well-controlled, partially controlled, and uncontrolled asthma in accordance with their clinical picture. Bronchial asthma in patients was diagnosed in line with recommendations indicated in the Global Initiative for Asthma: Global strategy for asthma management and prevention 2022 (GINA, 2022). At the time of recording, the volunteers had no pulmonary diseases or other diseases causing pathological changes in breath sounds.

Clinical examination of patients and respiratory sound recordings was performed at the Regional Children’s Clinical Hospital of Perm Krai (Perm, Russia). The examinations were carried out in accordance with the Declaration of Helsinki, adopted in June 1964 (Helsinki, Finland), revised in October 2000 (Edinburgh, Scotland), and approved by the Ethics Committee of the Perm State Medical University. A written consent was obtained from the examined persons (or from their parents or guardians when children were examined) in accordance with the Federal Law “The Fundamentals of the Legislation of the Russian Federation on the Protection of Citizens’ Health” from July 22, 1993, No. 5487-1.

No volunteers or patients smoked.

The database contains 1,371 recordings of respiratory sounds of patients and healthy volunteers aged between 18 months and 47 years. The distribution of recordings by gender, age, and disease is presented in Tables 1–3. The “Asthma” section of Table 3 shows the total number of patients with asthma at different levels of control. More than 70% of records are for boys/men (Table 1). Most patients participating in the study were between four and 20 years old (Table 2). In this study, the main emphasis was placed on development of computer-assisted methods of asthma diagnostics, and there are 1,113 recordings of asthmatic patients in the database. To verify the applicability of the proposed approach for development of differential diagnostic methods, respiratory sounds of patients suffering from other respiratory diseases were also recorded (Table 3). Diagnostics of these diseases was performed using a set of generally accepted studies.

We develop two diagnostic methods: diagnostics of respiratory illnesses using classification “sick/healthy” and differential diagnostics of asthma according to classification “asthmatic patient/non-asthmatic patient and healthy”. In the first case the healthy volunteers can be considered as a control group. In the second case a control group is formed by non-asthma patients and volunteers.

## Recording of Respiratory Sounds

Breath sounds were recorded in calm breathing at four points: near the oral cavity, above the trachea, on the chest (the second intercostal space on the right side), and at a point on the right side of the back (Table 4). For most of the examined subjects, including asthmatic patients with different states of the disease; the recordings were performed at several points. A greater part (about 60%) of recordings was performed near the trachea (Table 4). Choice of this point was conditioned by the following: (1) airflow in the trachea contains information about sounds in the lung as a whole; (2) noises caused by cardiac murmurs and muscular activity are weaker here than the ones at the points on the chest and back; (3) it enables registration of sounds both at the inbreath and the exbreath. Breath sounds were registered without interruption for several respiratory cycles, about 25 s. The minimum recording time was 16 s. This reduced the influence of random variations in sound intensity on the results of analysis.

**Table 1:** Gender distribution.

**Gender**	**Asthma**	**Healthy**	**Ill** (**no asthma)**	**Total records**
F	313	52	24	389
M	800	81	101	982
**Total**	1,113	133	125	1,371

**Table 2:** Age distribution.

	**0–2**	**2–4**	**4–13**	**13–20**	**20+**	**Total**
Asthma	14	8	515	516	60	1,113
Healthy	6	0	32	13	82	133
Ill (no asthma)	1	6	110	8	0	125
**Total records**	21	14	657	537	142	1,371

**Table 3:** Disease distribution.

**Disease**	**F**	**M**	**Total**
Asthma (partially controlled)	113	307	420
Asthma (exacerbation or/and uncontrolled)	53	131	184
Asthma (well-controlled)	77	166	243
Asthma (unspecified disease control)	70	196	266
Healthy	52	81	133
Relapsing obstructive bronchitis	13	84	97
Other lung diseases	11	17	28
**Total**	389	982	1,371

**Table 4:** Recording site distribution.

**Recording point**	Asthma^*^	**Healthy**	**Ill** **(no asthma)**	**Total records**
Second intercostal space	285	9	10	304
Chest from behind	256	0	12	268
Oral cavity	9	1	2	12
Trachea	563	123	101	787
**Total**	1,113	133	125	1,371

**Notes.**

* Asthma–number of records at all disease stages.

Respiratory sounds were registered and recorded using mobile phones and computer systems for sound recordings. The files containing respiratory sound recordings created with phones with both internal and external microphones were transferred to a cloud storage to create an anonymous database. Computer recordings were done using developed computer systems for respiratory sound recording (Furman et al., 2014; Furman et al., 2015; Furman et al., 2021; Furman et al., 2020). These computer systems contain external microphones, electronic phonendoscopes, and computer sound cards, as well as Adobe Audition software. The computer systems used to produce the recordings and the quality of the recordings meet requirements defined in Reicbert et al. (2009). All systems demonstrated high amplitude–frequency linearity in the range from 100 Hz to 3,000 Hz. The sampling rate was varied from 22 kHz to 96 kHz. Recordings were made in wav, mp3, and m4a formats. Our analysis of the recordings in different formats showed that the Fourier spectra of the recordings slightly differ in the frequency range we are interested in. The files in mp3 and m4a formats were converted to wav format.

All recordings were performed by physicians with special training to use registration systems and audio recording software. The physicians were also trained to visually control the quality of the recordings (oscillograms of the recordings were shown on the computer screen) and to analyze the visual presentation of the sound recordings. In addition to this the recording quality was assured with the help of the developed software.

## Methods of Analysis of Respiratory Sound Recordings

**Figure 1:**
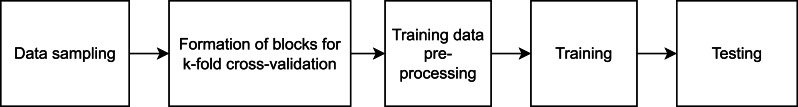
High-level diagram of the respiratory sound analysis workflow.

This study was carried out in accordance with the approval (approval code: 7/21, approval date:10.02.2021) received from the local research ethics committee at the Academician E.A. Wagner Perm State Medical University. In Tomita et al. (2019), a method of computer-assisted diagnostics of asthma that utilizes deep learning techniques has been developed. At the input layer, information regarding a patient, their anamnesis, results of biochemical analyses, and functional lung testing are introduced. We are proposing a method, whose sole input element is the audio file. The suggested method is based on visualization of respiratory sound recordings followed by training of a DenseNet model with k-fold cross-validation. The k-fold cross-validation approach is successfully used to analyze images (Han et al., 2021; Rodriguez, Perez & Lozano, 2010) including in methods of computer-assisted diagnostics based on computed tomography. As the result of creating different samples from our database, we have developed software to perform binary classification of two types “sick/healthy” and “asthmatic patient/non-asthmatic patient and healthy.” For the “sick/healthy” classification, by “sick” we mean that a patient was diagnosed with any of the respiratory diseases indicated in the “Database” section. The models trained to perform the aforementioned binary classifications and their subsequent use enable not only the diagnostics of bronchial asthma but preliminary differential diagnostics as well. The program workflow is presented in Fig. 1.

## Samples and Data Preprocessing

**Figure 2:**
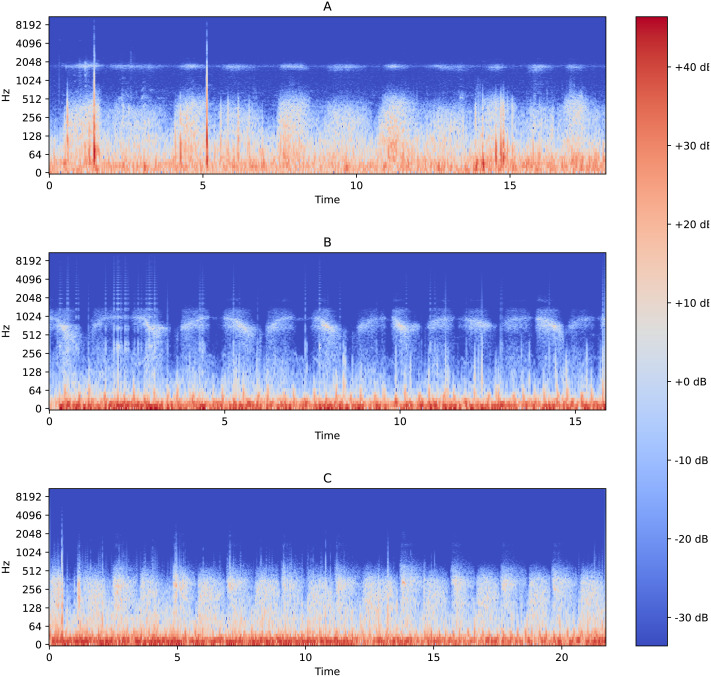
Fourier spectrograms of respiratory sound spectra in patients suffering from bronchial asthma (A) and (C) and a healthy volunteer (B).

**Figure 3:**
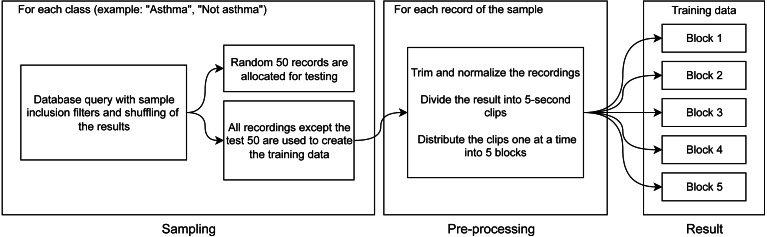
Diagram of training data preparation procedure.

The functionality of performing binary classification and processing of audio records is coupled in the same piece of software. Characteristic Fourier spectrograms of respiratory sound spectra of two asthmatic patients and a healthy volunteer are plotted in Fig. 2. In the first stage, the database is used to filter and gather samples and groups in line with the procedure illustrated in Fig. 3. To produce equally weighted samples from a group containing a larger number of files, the recordings were randomly selected equal to the number of files in the smaller group. For example, for the binary classification “sick/healthy,” the database contains 1,238 records of patients and 133 records of healthy volunteers. In the groups, all 133 recordings of healthy volunteers and 133 randomly selected recordings of patients are used. From each group, 50 recordings were randomly allocated to be used for testing, and the remaining recordings were used for training the model. Testing was also performed on all patient recordings that were not in any of the training or testing samples.

The selected files are subject to preprocessing, during which the opening and end portions (2 s each) that often contain extraneous noises or/and an absent signal are removed from the records. Extraneous or random extremes in the recorded signals are also removed, and the digital signal representation is normalized to a range from −1 to 1. The processed files are divided into 5-second clips with 50% overlapping. From the final 5-second clips, five blocks for k-fold cross-validation are assembled (Fig. 3).

Note that the clips are allocated to the blocks sequentially, which ensures that clips from the same file end up in different blocks. This ensures that each of the blocks will be representative. The described division of recordings into clips allows us not only to unify the analyzed records but also to increase (augment) the sample by modifying the existing data.

The next stage is illustrated in Fig. 3: for each clip, we make three spectrograms of mel-frequency cepstral coefficients calculated with different sliding window widths using the discrete Fourier transformation. The window widths for the three cepstrograms are 25 ms, 100 ms, and 175 ms. The window widths were chosen empirically from the widths that deliver the best results. These windows are consistent with the duration (from 80 ms to 250 ms Sovijarvi et al., 2000; Bahoura & Lu, 2006) of wheezing breathing of asthma patients.

Spectrograms were made using a function included in the PyTorch library (*MelSpectrogram*) (Paszke et al., 2019). Use of such spectrograms for asthma diagnostics was proposed in a number of works (Yu et al., 2013; Reichert et al., 2008; Shirazi et al., 2012; Taplidou & Hadjileontiadis, 2007; Kim et al., 2021). In these works, the physician was offered the choice of performing the diagnostics by visual signs of the disease available on a spectrogram or using computer-aided analysis of amplitude–frequency dependencies. This visual method is subjective and in certain cases, diagnostics can be difficult and lead to an incorrect result (see Appendix). Computer-assisted analysis somewhat enhances the accuracy of diagnostics.

## Training the Models

To implement binary classification of the clips, we chose the DenseNet201 model originally proposed in the article “Densely Connected Convolutional Networks” (Huang et al., 2018). The choice of this model is conditioned by the following factors. On the one hand, this model is more efficient than other models in classifying audio records (Palanisamy, Singhania & Yao, 2020). Its architecture was successfully used for COVID-19 diagnostics based on analysis of audio recordings (Laguarta, Hueto & Subirana, 2020). On the other hand, for the architecture used, there are pre-trained states for classifying audio recordings that were created by training using large databases of recordings, in particular, ESC (Piczak, 2015), that enables us to utilize the transfer learning approach. Transfer learning enables the use of preliminary trained statistical models to train new models using only small amounts of new data of the same type (Pratt, 1992; Han et al., 2021). To apply the method of transfer learning for our models during initialization, we used a state of the model pre-trained on ESC.

**Figure 4:**
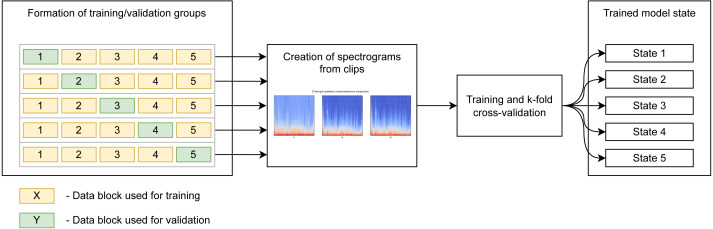
Preparation of spectrogram for learning.

We use k-fold cross-validation of five blocks containing the same number of records. The model is trained five times independently, sequentially, using four blocks for training and using the fifth block for validation. This gives an 80/20 proportion in the distribution of data into training/validation groups, and we end up with five independent states of the model for testing. The diagram (Fig. 4) shows the use of blocks to form the training and validation samples in different iterations of training. It should be noted that learning, validation, and subsequent testing were implemented using recordings captured at all points.

During training, each of the groups with training and validation data went through the model 60 times in both directions (60 epochs of training). The number of epochs was limited, in order to avoid overfitting the models.

We select the state of the model that has shown the best validation result after the learning dynamics (increasing accuracy with each subsequent training epoch) is reduced. This is done to simultaneously obtain high accuracy and prevent the model’s overfitting.

**Figure 5:**
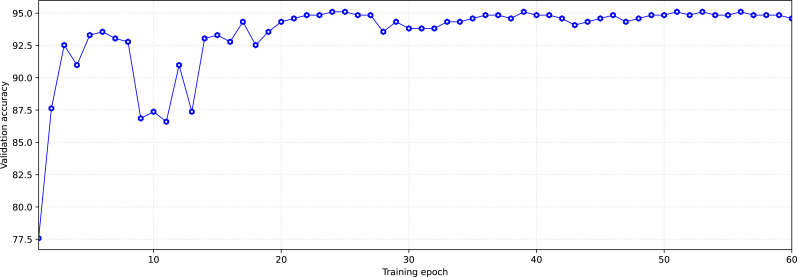
Typical dynamics of training model at the example “asthmatic patient/non-asthmatic patient and healthy”.

**Figure 6:**
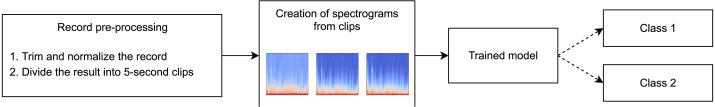
Prediction (inference) procedure.

As an example, Fig. 5 shows the dynamics of model learning. The state reached at the 59-th training epoch for the learning/validation group was chosen as the trained model. This state was chosen based on two criteria. The first criterion is high accuracy in validation, the second one is the later epoch (in order not to get insufficiently trained models, the states reached at epochs with numbers greater than 40 were chosen). After the training, we tested the models trained on each training/validation group using test data. To test, data from the test sample was provided as the model input so that the model would perform a prediction (inference) of the appropriate class (Fig. 6).

As an example, Table 5 shows the results of model testing for the classification of respiratory sound recordings into ”asthmatic patient/non-asthmatic patient and healthy” classes. Sensitivity *S*_*e*_ and specificity *S*_*p*_ were used as the accuracy criteria for the model:

\begin{eqnarray*}{S}_{e}& = \frac{{T}_{p}}{{T}_{p}+{F}_{p}} 100\text{%} \end{eqnarray*}

\begin{eqnarray*}{S}_{p}& = \frac{{T}_{h}}{{T}_{h}+{F}_{h}} 100\text{%} \end{eqnarray*}

where *T*_*p*_ and *T*_*h*_–numbers of correctly diagnosed patients and healthy persons, respectively; *F*_*p*_ and *F*_*h*_ –numbers of mistakenly diagnosed patients as healthy and healthy as ill, respectively.

**Table 5:** Results of model testing to classify asthmatic patient/non-asthmatic patient and healthy.

	**Asthma P**	**Asthma FN**	**No asthma P**	**No asthma FN**	**Sensitivity**	**Specificity**
Group 1	46	4	40	10	92%	80%
Group 2	45	5	38	12	90%	76%
Group 3	45	5	40	10	90%	80%
Group 4	46	4	40	10	92%	80%
Group 5	46	4	41	9	92%	82%

**Notes.**

Here, P –correct diagnostics (positive classification), FN –incorrect diagnostics (false-negative classification).

Since we use the 5-block cross-validation as a result of training, we obtain five states of the selected model. All five model states obtained give close results. Out of them, we chose the model that shows the best results for further use. In this example, it is model number 5.

## Diagnostics

The trained models were tested using recordings contained in the sample that did not participate in either training or validation. Testing was performed for two types of binary classifications (diagnoses): “sick/healthy” and “asthmatic patient/non-asthmatic patient and healthy,” using balanced test samples of 50 randomly selected recordings of each type.

Here, the method accuracy is determined by the formula:

\begin{eqnarray*}A= \frac{{T}_{p}+{T}_{h}}{{T}_{p}+{F}_{p}+{T}_{h}+{F}_{h}} 100\text{%} \end{eqnarray*}

and Youden’s index is calculated using the formula:

\begin{eqnarray*}J= \left( \frac{{T}_{p}}{{T}_{p}+{F}_{p}} + \frac{{T}_{h}}{{T}_{h}+{F}_{h}} \right) . \end{eqnarray*}

In testing, the sensitivity and specificity of both classifications are almost the same as the values obtained in training and validating the models (Tables 5 and 6).

**Table 6:** Results of predictions/inferences (diagnostics) for test samples.

Classification type	Validation accuracy	Sensitivity	Specificity	Accuracy	Youden’s index
Sick/healthy	96%	88%	86%	87%	0.74
Asthmatic patient/Non-asthmatic patient and healthy	94%	92%	82%	87%	0.74

The proposed models allow one to diagnose and classify patients as respiratory patients, using the criterion “sick/healthy,” as well as “to separate” the asthmatic patients from the group of non-asthmatic patients and healthy people. Consistent use of developed models may provide the basis for differential diagnostics. We differentially diagnosed asthmatic patients using the “asthmatic patient/non-asthmatic patient and healthy” classifier and applied it to 941 records of asthmatic patients from the database, who did not participate in training and model testing. The sensitivity of this differential diagnostics was 93%.

## Discussion and Conclusion

We have shown that the proposed methods demonstrate high sensitivity and specificity for diagnostics of bronchial asthma and can provide the basis for the development of methods of computer-assisted differential diagnostics. The method is based on representation of a sound recording as a spectrogram image, which is being classified as one of two classes (binary classification) using deep learning techniques. This approach makes it possible to determine signs of the disease (characteristic pathological changes in respiratory sounds), which cannot be diagnosed by a human (see Appendix).

Using the proposed method, we developed two computer programs (software) to diagnose respiratory patients and, differentially, asthmatic patients. Applied to small test samples (groups), 50/50, the sensitivity, specificity, and accuracy were 88%, 86%, and 87%, respectively, for the “sick/healthy” classification. These values for the “asthmatic patient/non-asthmatic patient and healthy” classifier were as follows: sensitivity for diagnosing asthmatic disease was 92% with specificity of 82% and accuracy of 87% (Table 5).

The achieved diagnostic indicators exceed those of traditional diagnostic methods. Clinical auscultation is an inexpensive, safe, easy-to-perform diagnostic tool. Lung auscultation by a physician is a subjective method with variability and depends on several factors, including the qualifications and experience of the physician. The average percentage of correct detections of wheezing by non-pulmonologists is 60%; this percentage for pulmonologists is somewhat higher (Hafke-Dys et al., 2019). Note that during auscultation a physician determines pathological sounds, such as wheezing, which can be caused by various illnesses (GINA, 2022; Furman et al., 2020; Sovijarvi et al., 2000).

Analysis of the lungs’ functional state is recommended by the GINA (2022) to be used for bronchial asthma diagnostics. These exams reflect documented expiratory airflow limitation caused by the illness. For spirometry (Schneider et al., 2009) sensitivity is 29% (when performed by high-level specialists and with active cooperation of the patient it can reach 39.8%), and specificity of 90%. The change of peak flow in the SAPALDIA study (Künzli et al., 1999) is characterized by the following: sensitivity of 40% and specificity of 83%; when diagnosing the disease in children (Valadares et al., 2013): sensitivity is 31% and specificity –90%.

A method for classifying pathological respiratory sounds and models (neural networks) built on different architectures (VGG16, VGG19, Inception V3, DenseNet201, ResNet50, and ResNet101) was analyzed in Kim et al. (2021). The best model distinguished pathological sounds, without performing disease diagnostics, with a reliability of 86.5%.

The accuracy of computer-assisted diagnostics of pathological sounds and their classification by type (wheezing, rhonchi, crackles) exceeded the accuracy of the physician’s diagnosis. A computer-based method for diagnosing BA (Gelman et al., 2021) based on machine learning and comparison of respiratory sounds of asthmatic patients and healthy volunteers demonstrates similar values of sensitivity of 89.3%; reliability of 86%, accuracy of about 88%, and Youden’s index of 0.753.

The “asthmatic patient/non-asthmatic patient and healthy” classifier that we developed on a rather large database of 941 asthmatic patient recordings demonstrated high sensitivity of 93% for differential diagnostics.

Relatively small databases were used to train, validate, and test the models. Segmentation of audio recordings and transfer learning methods were used to increase their diagnostic performance. Improving the quality of the models is directly related to increasing the amount of data in the database.

In pulmonary diseases, pathological processes and the physiological changes provoked by them are reflected in the character of respiratory sounds. The computer-assisted diagnostics methods developed previously that utilize the Fourier spectrum analysis or other sound wave metrics attempt to link disease diagnostics with the analysis of biological, physiological, and clinical processes in the patient’s body (Furman et al., 2014; Furman et al., 2015; Furman et al., 2021; Furman et al., 2020; Gelman et al., 2021). Machine learning and deep learning methods are designed to divide data into certain categories according to identified common attributes that cannot always be clearly matched to body processes, and to statistically analyze the signals. This leads to the fact that the computer can distinguish signs that are not evident to a human observer, and computer-assisted diagnostic methods based on machine learning techniques can be more accurate than methods that identify pathological characteristics of sounds. So, we may assume that the proposed methods can be used not only for registration of characteristics of asthmatic pathological sounds but also to assist in the diagnostics of asthma.

The method we developed possesses a certain universality: it classifies recordings made at different points (second intercostal space, chest from behind, oral cavity, and trachea) in patients at different stages of asthmatic disease (exacerbation, well-controlled, partially controlled, and uncontrolled asthma) as well as recordings made using different devices, including smartphones, and encoded in different formats wav, mp3, m3a. Once trained, the software makes it possible to diagnose bronchial asthma with high reliability regardless of the gender and age of the patient, stage of the disease, and the point at which the sounds are recorded. This opens a way to use the proposed method in telemedicine. One of potential areas of future development may be its adaption to asthma exacerbation diagnostics.

To conclude, the proposed method of computer diagnostics based on the analysis of respiratory sounds enables diagnostics of bronchial asthma with high reliability irrespective of the patient’s gender and age, stage of the disease, and the point, at which the sounds are recorded. The method is based on visualization of audio recordings and uses deep learning techniques for their classification. We developed methods of binary classification of respiratory diseases, which demonstrate high sensitivity and specificity in diagnostics of bronchial asthma.

The methods may serve as the basis for development of differential computer-assisted diagnostics.

Bronchial asthma diagnostics is based on a comprehensive examination of a patient, including lung auscultation and lung function assessment. The proposed method can be used as an additional screening method for preclinical bronchial asthma diagnostics and serve as a basis for the development of computer-aided monitoring methods, including remote monitoring (telemedicine) of patient’s condition and real-time assessment of treatment effectiveness.

##  Supplemental Information

10.7717/peerj-cs.1173/supp-1 Supplemental Information 1 Metadata of the audio files database The metadata of the audio files database that was used and 4 sample audio files to use with the example notebooks. Pre-trained snapshots of DenseNet models required to run the inference are available for download from public Google Drive links.

## Appendix

The results of lung auscultations performed by physicians are largely subjective, depending on the physician’s experience (Kim et al., 2021), and the human ear’s perception of pathological sounds is limited both in frequency and in their level against other noises. Computer methods for diagnosing pulmonary diseases are devoid of subjectivity and make it possible to analyze the changes in respiratory sounds that cannot be detected by a human ear. One such method is visualization imaging of respiratory sounds (Li et al., 2016). For example, the Fourier spectrum of respiratory sounds of asthmatic patients contains areas of frequencies in which the amplitudes of harmonics significantly exceed the amplitudes in the spectrum of healthy people (see, for example, Fig. 4 in Furman et al., 2014). A time-periodic manifestation of such amplification is observed. The duration of such amplifications is about 200 ms (see, for example, Fig. 5 in Furman et al., 2014). This makes it possible to visually distinguish between the respiratory sounds of ill and healthy people.

However, diagnostics based on visualization of respiratory sounds has similar drawbacks: it is subjective and limited to the perception of a human eye. To illustrate this, characteristic Fourier spectrograms of respiratory sound spectra of two asthmatic patients and a healthy volunteer are plotted in Fig. 2. In the first stage, the respiratory sound recordings were represented in a time-frequency domain using a discrete Fourier transform for short overlapping time intervals. We used a sliding window containing 2,048 recording time points, and the window shift step was chosen to be 512 points. Further, the squares of harmonic amplitudes as a function of time and frequency were represented in decibels. The functions stft and amplitude_to_db from the librosa library (McFee et al., 2021) were used to perform these operations.

Two types of spectrograms of patients can be seen clearly: spectrogram (Fig. 2A) with clearly pronounced periodic sound amplification (an increase of harmonic amplitudes) compared to the spectrogram of a healthy person (Fig. 2B) in the frequency range from 65 Hz to 510 Hz and also in a narrow frequency range around 2,500 Hz (Fig. 2A). In the frequency range from 65 Hz to 510 Hz, the duration of harmonic amplitude increase is approximately 1 s. Within these time intervals, additional short-term (∼150 ms) sound amplifications are observed. At high frequencies (∼2,500 Hz), the duration of periodic sound amplifications is approximately 1.2 s.

The second type of spectrograms of patients (Fig. 2C) is characterized by periodic amplification of sounds in the frequency range from 100 Hz to 500 Hz. The duration of such amplifications is ∼200 msec. The same amplifications, but less pronounced, are also observed on the spectrogram of a healthy person (Fig. 1B). Taking into account that sounds at frequencies less than 100 Hz are mainly determined by noises of the heart and chest muscles (Furman et al., 2014; Furman et al., 2015; Furman et al., 2021), it is very difficult to diagnose the disease visually by the spectrogram in Fig. 1C.

## Additional Information and Declarations

### Data Availability

The following information was supplied regarding data availability: The raw data is available in the Supplementary Files.
